# Decreased Resting-State Functional Connectivity of Periaqueductal Gray in Temporal Lobe Epilepsy Comorbid With Migraine

**DOI:** 10.3389/fneur.2021.636202

**Published:** 2021-05-26

**Authors:** Long Wang, Xin-Ting Cai, Mei-Dan Zu, Juan Zhang, Zi-Ru Deng, Yu Wang

**Affiliations:** ^1^Department of Neurology, The First Affiliated Hospital of Anhui Medical University, Hefei, China; ^2^Department of Neurology, The Second People Hospital of Hefei, Hefei, China

**Keywords:** epilepsy, migraine, comorbidity, periaqueductal gray, pedunculopontine nucleus, functional magnetic resonance imaging

## Abstract

**Objective:** Patients with temporal lobe epilepsy (TLE) are at high risk for having a comorbid condition of migraine, and these two common diseases are proposed to have some shared pathophysiological mechanisms. Our recent study indicated the dysfunction of periaqueductal gray (PAG), a key pain-modulating structure, contributes to the development of pain hypersensitivity and epileptogenesis in epilepsy. This study is to investigate the functional connectivity of PAG network in epilepsy comorbid with migraine.

**Methods:** Thirty-two patients with TLE, including 16 epilepsy patients without migraine (EwoM) and 16 epilepsy patients with comorbid migraine (EwM), and 14 matched healthy controls (HCs) were recruited and underwent resting functional magnetic resonance imaging (fMRI) scans to measure the resting-state functional connectivity (RsFC) of PAG network. The frequency and severity of migraine attacks were assessed using the Migraine Disability Assessment Questionnaire (MIDAS) and Visual Analog Scale/Score (VAS). In animal experiments, FluoroGold (FG), a retrograde tracing agent, was injected into PPN and its fluorescence detected in vlPAG to trace the neuronal projection from vlPAG to PPN. FG traced neuron number was used to evaluate the neural transmission activity of vlPAG-PPN pathway. The data were processed and analyzed using DPARSF and SPSS17.0 software. Based on the RsFC finding, the excitatory transmission of PAG and the associated brain structure was studied via retrograde tracing in combination with immunohistochemical labeling of excitatory neurons.

**Results:** Compared to HCs group, the RsFC between PAG and the left pedunculopontine nucleus (PPN), between PAG and the corpus callosum (CC), was decreased both in EwoM and EwM group, while the RsFC between PAG and the right PPN was increased only in EwoM group but not in EwM group. Compared to EwoM group, the RsFC between PAG and the right PPN was decreased in EwM group. Furthermore, the RsFC between PAG and PPN was negatively correlated with the frequency and severity of migraine attacks. In animal study, a seizure stimulation induced excitatory transmission from PAG to PPN was decreased in rats with chronic epilepsy as compared to that in normal control rats.

**Conclusion:** The comorbidity of epilepsy and migraine is associated with the decreased RsFC between PAG and PPN.

## Introduction

Temporal lobe epilepsy (TLE) is the most common form of epilepsy which is often characterized by drug resistance (1). Imaging evidences have shown that brain network abnormalities in TLE are not limited within the epileptogenic focus but rather throughout the whole brain (2, 3). TLE is at high risk for having comorbid condition of migraine, and these two common diseases are proposed to have some shared pathophysiological mechanisms (4, 5). Moreover, comorbid migraine may be a predictor of medically refractory epilepsy, indicating its impact on epilepsy (6). Therefore, a better understanding of the neurophysiological mechanism related to the comorbidity of epilepsy and migraine is helpful to develop more effective treatments.

The midbrain periaqueductal gray (PAG) is a group of structural and functional heterogeneous nuclei, which is divided into four independent but interconnected subregions: ventrolateral PAG (vlPAG), lateral PAG (lPAG), dorsomedial PAG (dmPAG), and dorsolateral PAG (dlPAG) based on cytoarchitectonics, histochemistry, and neuroimaging studies (7, 8). As a functional heterogeneous nuclei, PAG is a key brainstem structure for modulation of multiple neurophysiological functions including pain (9, 10), emotion (11), respiration, and cardiovascular activity (12). Especially, PAG is the primary center of the descending pain inhibition system involved in regulation of both endogenous pain and trigeminovascular pathway pain (9, 10). Structural and functional alterations of PAG have been identified in migraine (13–17), and it has been proposed that PAG dysfunction results in trigeminovascular neuron hyperexcitability leading to migraine headache (18). This is further supported by multiple clinical case descriptions that PAG lesion, especially ventrolateral PAG (vlPAG) lesion, can initiate migraine-like headache attacks (19–21). In fact, the dysfunction of PAG has now been considered a “generator” of migraine headache (22, 23). Experimental studies have demonstrated vlPAG is critically involved in the development of post-ictal antinociception in animal study as well as in that of pain sensitivity decrement during the interphase of chronic epilepsy in human study (24, 25). In addition, we have recently demonstrated that vlPAG is not only involved in pain regulation in epilepsy but also in the development of epileptogenesis (26).

Another upper brainstem structure, pedunculopontine nucleus (PPN), which has functional afferent as well as efferent connections with multiple brain structures including vlPAG and structures outside of brainstem (27), is also closely related to the post-ictal antinociception (28, 29). Some studies have also demonstrated that stimulation of PPN has anti-epileptic effects, though this method of PPN stimulation has not been accepted in clinical practice (30, 31).

Taken together, it is clear that vlPAG and PPN are both involved in modulation of pain and epilepsy. Thus, we speculate that there may be an abnormal functional connection between vlPAG and PPN in epilepsy patients with comorbid migraine. However, vlPAG is difficult to be completely separated from PAG in neuroimaging because of the complexity and particularity of PAG structure, so we conducted the current study with a whole brain functional connectivity (FC) selecting PAG as the region of interest (ROI). And we will preliminarily verify the findings of the clinical imaging study through animal experiments.

## Materials and Methods

### Patient Study

#### Participants

A total of 32 TLE patients with bilateral tonic-clonic seizures were recruited from the outpatient department at the First Affiliated Hospital of Anhui Medical University in Hefei from August 2019 to January 2020. Sixteen TLE patients were comorbid with migraine (EwM group) and 16 without migraine (EwoM group). The inclusion criteria were as follows: (1) patients with epilepsy of only bilateral tonic-clonic seizures; (2) seizure types of bilateral tonic-clonic seizures were established according to the classification of the International League Against Epilepsy (ILAE) (32–34); (3) all patients accepted 3.0 T magnetic resonance imaging (MRI) examination to exclude intracranial tumor, changes after cerebrovascular disease, post traumatic changes, and other conditions associated with brain structure alterations; (4) Mini-mental State Examination (MMSE) score ≥24 points (35); (5) all patients were right-handed. The exclusion criteria were as follows: (1) patients with a history of other neurological, mental, or chronic diseases, such as schizophrenia, diabetes, etc.; (2) pregnant and nursing women; (3) patients with a history of metal dentures or implants; (4) patients who experienced generalized bilateral tonic-clonic seizure within 1 week prior to fMRI scanning; (5) excessive motion (>2.5 mm, 2.5°) during fMRI scanning. All epilepsy patients with or without migraine were on anti-epileptic treatment with monotherapy or combination therapy. All epilepsy patients with migraine had never accepted special treatment for migraine prophylaxis though some antiepileptic drugs may have anti-migraine effects. Fourteen right-handed healthy participants matched with the epilepsy group in age, sex, and educational level were recruited as healthy controls (HCs group).

#### Clinical Assessment and Analysis

Clinical information including demographic data, education level, past history of medical, and using of anti-depressive drugs were collected from all patients. Mini-mental State Examination (MMSE) and Hamilton Depression Rating Scale (HDRS) were, respectively used to evaluate the cognitive function and depressive symptoms of the participants (35, 36). Migraine head pain severity was measured by means of a Visual Analog Scale (VAS) with 0 indicating no head pain and 10 indicating unbearable head pain (37). The statistical analyses of the clinical data were conducted with SPSS version 16.0 (SPSS lnc., Chicago, USA), with a significant threshold of *P* < 0.05.

#### Neuroimaging Data Acquisition

The resting-state functional magnetic resonance imaging (rs-fMRI) was obtained using a 3.0 T MRI scanner (Discovery GE750w; GE Healthcare, Buckinghamshire, UK) from the University of Science and Technology of China. All participants were required to keep their eyes closed without either thinking about anything or moving the body during scanning. MR imaging system was composed of 217 echo-planar imaging sequences with the following parameters: repetition time/echo time (TR/TE): 2,000/30 ms; flip angle: 90°; matrix size: 64 × 64; field of view (FOV): 192 × 192 mm^2^; slice thickness: 3 mm; 46 continuous slices (voxel size: 3 × 3 × 3 mm^3^). T1-weighted anatomic images with 188 slices were also obtained in sagittal orientation with the following parameters: TR: 8.16 ms; TE: 3.18 ms; flip angle: 12°; FOV: 256 × 256 mm^2^; slice thickness: 1 mm; voxel size: 1 × 1 × 1 mm^3^.

#### rs-fMRI Preprocessing

Functional image preprocessing was carried out using the Data Processing Assistant for rs-fMRI toolkit software package based on Statistical Parametric Mapping software (SPM12; www.fil.ion.ucl.ac.uk/spm) and rs-fMRI Imaging Toolkit (38, 39). Software was preprocessed in the following steps: conversion of the DICOM data to NIFTI images; removal of the first 10 time points to exclude the influence of unstable longitudinal magnetization; slice timing correction; motion correction; realignment to respective structural image; nuisance regressors, spatial normalization based on the segmentation of structural images, spatial smoothing (Gaussian kernel, FWHM = 4 mm), and filtering temporal band pass (0.01–0.1 Hz).

#### RsFC Processing and Analysis

**Figure 1:**
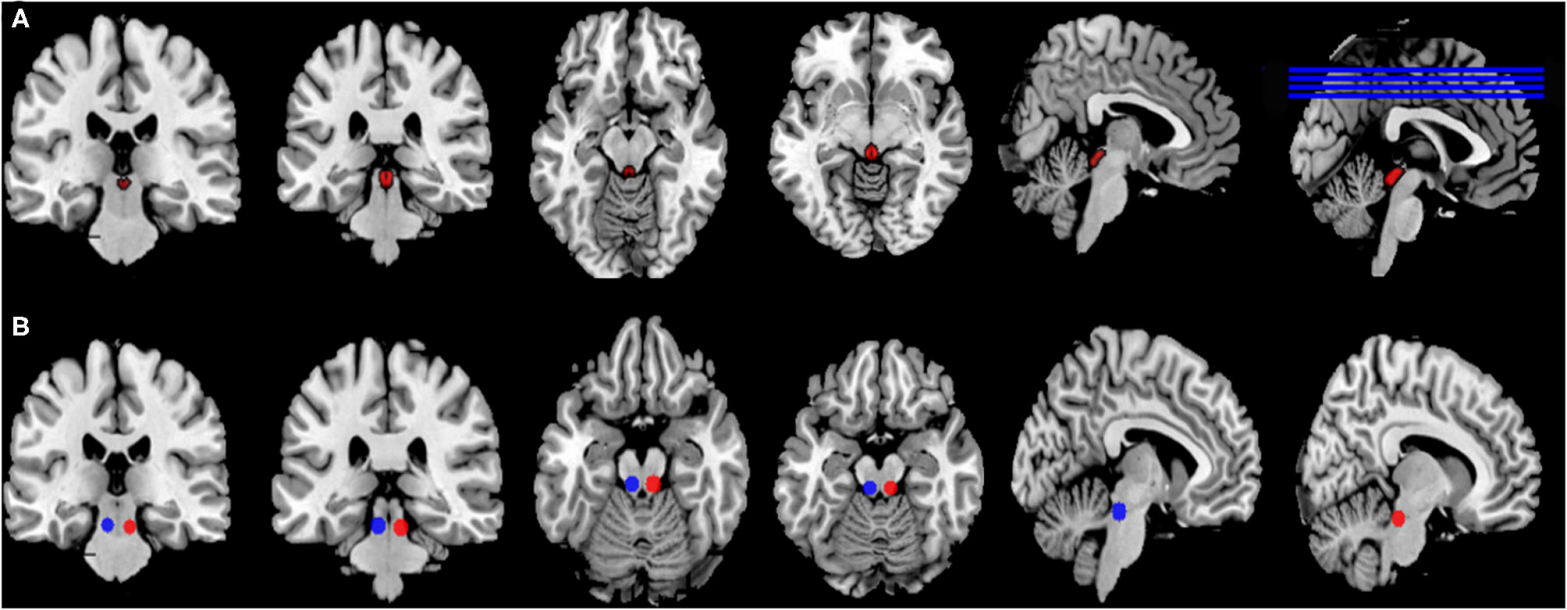
Location of seeds and ROI. **(A)** The ROI of the PAG was obtained from the Keuken and Forstmann's 7T atlas that provides ROIs obtained from high-resolution MP2RAGE and FLASH scans warped in MNI space (Keuken and Forstmann, 2015), Available at https://www.nitrc.org/project cts/atag/; **(B)** The ROI of double PPN was generated by placing two 5 mm radius spheres with centers at coordinates [±6.4, −27, −15] in MNI standard space.

The mask of the PAG was obtained from the Cacciola and Keuken's 7T atlas that provides ROIs obtained from high-resolution MP2RAGE and FLASH scans warped in MNI space available at www.nitrc.org/projects/atag/ (40, 41) (Figure 1A). The location of the PPN was generated by placing two 5 mm radius spheres with centers at coordinates [±6.4, −27, −15] in MNI standard space (42, 43) (Figure 1B). To obtain the value of RsFC, the mean time series of PAG was extracted and then used to calculate the Pearson's correlations (r) with voxels in the remaining brain in each participant. The Fisher's z r-to-z transformation was applied to improve the normality, and then the results were displayed with an RsFC map for each participant. To explore RsFC differences among 3 groups, independent samples *t*-tests and one-way analysis of variance (ANOVA) were performed, respectively using DPABI software within a mask of whole brain gray matter. Moreover, clinical data including HDRS, medications, and seizure duration and frequency may have influence on RsFC results (44) as these confounding factors were statistically different between groups (Table 1). Thus, HDRS, medications, and seizure duration and frequency were used as covariables for calculation. *Post-hoc* analyses were conducted using Gaussian Random Field method (GRF) with the significance of voxel level set at *P* < 0.001, and that of cluster level set at *P* < 0.05, two-tailed.

**Table 1:** Demographic and clinical characteristics of the participants.

**Characteristics**	**HCs (*n* = 14)**	**EwoM (*n* = 16)**	**EwM (*n* = 16)**	***P***
Gender (M/F)	7/7	7/9	6/10	0.774^*^
Age, years, mean (SD)	25.6 (4.6)	28.8 (5.9)	25.3 (6.3)	0.169
Education, years, mean (SD)	12.6 (3.8)	14.0 (4.0)	11.9 (3.4)	0.276
MMSE, scores, mean (SD)	29.4 (0.9)	29.0 (1.8)	28.3 (1.9)	0.178
HDRS, scores, mean (SD)	1.4 (0.9)	2.5 (1.3)	7.6 (3.7)	<0.001
Epilepsy duration, month, mean (SD)	-	81.3 (53.7)	75.3 (57.8)	0.766
Mean seizure frequency, times/year, mean (SD)		2.3 (1.8)	4.8 (2.7)	0.005
Mean migraine frequency, days/month, mean (SD)		-	1.5 (0.5)	
**Form of migraine**				
IIM (patients number)			7	
PIM		-	12	
MwA		-	1	
MwoA		-	15	
VAS, scores, mean (SD)		-	4.5 (1.4)	
**Medications**				
Monotherapy (patients number)		12	6	0.033^*^
Drug combination		4	10	

*SD, standard deviation; HCs, healthy controls; EwoM, epilepsy without migraine; EwM, epilepsy with comorbid migraine; MMSE, Mini-mental State Examination; HDRS, Hamilton depression rating scale; IIM, inter-ictal migraine; PIM, post ictal migraine; MwA, migraine with aura; MwoA, migraine without aura; VAS, visual analog scale/score; n, number. Medications presents antiepileptic drugs*.

* *chi-square fisher exact test*.

#### Correlation Analysis

The RsFC of the PAG with corresponding significant areas were extracted and then Pearson's correlation analysis was conducted to detect the relationship between the values of RsFC and seizure events. The correlation analysis was performed by SPSS version 16.0 with a significant threshold of *P* < 0.05.

### Animal Study

Based on the functional imaging findings that the FC of PAG-PPN and PAG-CC were decreased in epilepsy patients, the animal study is aimed to address the alterations of the neural transmission from vlPAG to PPN in epileptic rats, using retrograde tracing and fluorescence immunohistochemistry.

#### Experimental Animals

Male Sprague-Dawley (SD) rats aged 4–5 weeks and weighed between 80 and 110 g, from the Animal Center of Anhui Medical University, were used for the experiment. The animals were kept in a temperature-controlled (22 ± 1°C) room with free access to food and water. The room was maintained on a light and dark cycle (12 h: 12 h, light from 6:00 to 18:00) and kept air humidity around 50%. The research complied with the national legislation on the Care and Use of Animals.

#### Experimental Design and Group Division

To investigate the activity of neural transmission from vlPAG to PPN, a retrograde tracing agent, FluoroGold (FG, Fluorochrome, USA), was injected into PPN and the traced neurons will be visualized in vlPAG. The FG-traced neuron number within vlPAG will be used to identify the neural transmission activity of vlPAG-PPN pathway. This FG retrograde tracing method has been widely used to study the activity of neuronal connections between different parts within the brain associated with distinct functions (45–47). For comparison of the neural transmission activity of vlPAG-PPN pathway between normal rats and chronic epileptic rats, seizure stimulation activating vlPAG-PPN pathway should be similar in severity and duration. Thus, status epilepticus (SE) was evoked with pilocarpine injection in naïve rats (Pilo group) as well as in rats with chronic epilepsy (1 month after SE induction) developed after SE induced by pre-treatment of pilocarpine injection (SE+Pilo group). In Pilo group, rats were stereotactically injected with FG to PPN 2 days before SE induction. In SE+Pilo group, rats were firstly induced with SE by plocarpine, and 28 days later, the rats were stereotactically injected with FG to PPN, and then, 2 days after FG injection, re-injected with pilocarpine to evoke a second SE as seizure stimulation. One hour after SE induction, i.e., seizure stimulation, rats were sacrificed for immunohistochemical staining.

#### Induction of SE

As previously described (48, 49), rats were firstly pre-treated with scopolamine methylbromide [1 mg/kg, intraperitoneal (i.p.), Sigma, USA] to prevent peripheral cholinergic effects caused by pilocarpine. Thirty minutes later, rats were then injected with pilocarpine (hydrochloride) dissolved in physiological saline (350 mg/kg/2 ml, i.p., Cayman, USA) to induce SE. For some rats, re-injections of 0.2 ml pilocarpine solution might be needed with an interval of 15 min. Behavioral seizures were recorded using previous five-stage scale modified mildly from Racine's scale (50): briefly, stage 1, face and vibrissae twitching, ear rubbing with forepaws, chewing; stage 2, head nodding or with unilateral limb clonus; stage 3, bilateral limb clonus, mild whole body convulsions; stage 4, lockjaw, rearing, whole body convulsions with tail hypertension; and stage 5, rearing with whole body convulsions and falling down with whole body rigidity. SE onset was defined as stage 3 or greater seizures that developed to repeated or prolonged behavioral seizures. Diazepam (10 mg/kg, i.p.) was administrated 60 min after SE induction to terminate convulsion. The duration of SE (seizure stimulation) in Pilo group rats was the same as that in SE+Pilo group rats as the seizures were terminated with diazepam when the SE duration reached 1 h. The stimulating seizure severity in Pilo group rats was close to that in SE+Pilo group rats as it reached SE in both groups.

#### Stereotactic Injection of FG

Rats were anesthetized by intraperitoneal injection of a mixed solution of 2% sodium pentobarbital (80 mg·kg^−1^) and xylazine (10 mg·kg^−1^). Fully anesthetized animals with regular respiratory rate were mounted on a stereotactic apparatus (RWD Life Science, China). A rostro-caudal incision was made in the skull skin after shearing hairs and full disinfecting with 1% iodophor. To expose bregma and posterior fontanelle completely, swabs were used to dip medical H_2_O_2_ to wipe soft tissues slightly. After adjusting bregma and posterior fontanelle to the same horizontal level, a hole was drilled and microinjection syringe was vertically introduced using the following coordinates based on Paxinos and Watson's rat brain in the stereotactic coordinates atlas: anteroposterior (AP), 0.2 mm; mediolateral (ML), 2.0 mm; and dorsoventral (DV), 7.0 mm to reach the PPN (28, 51). FG (Fluorochrome, USA) dissolved in physiological saline (4%) was unilaterally injected into the PPN at a rate of 0.25 μl per minute for 2 min using microinjection syringe fixed to the stereotaxic frame. The microinjection syringe remained there for 1 min to ensure FG was fully absorbed and then slowly pulled out. An intramuscular injection of penicillin (30 U/kg) was conducted to prevent infection after skull skin was sutured.

#### Tissue Preparation

One hour after SE (second SE for SE+Pilo group rats) was terminated, rats were anesthetized with pentobarbital (80 mg·kg^−1^) and xylazine (10 mg·kg^−1^) and perfused with 250 ml 0.9% physiological saline followed by 200 ml 4% paraformaldehyde in PBS (pH 7.4). Next, the brain was removed immediately, fixed in 4% paraformaldehyde at 4°C overnight, dehydrated in 30% sucrose at 4°C, and then quick frozen on dry ice and stored at −80°C. Serial coronal sections (10 μm) containing the vlPAG were obtained by a cryostat (CM 3050S, Leica, Germany). Each section has an interval of about 70 μm, and six sections taken from each rat were attached to poly-lysine-coated slides for immunofluorescence study.

#### Fluorescence Immunohistochemistry

Sections were incubated with 0.3% Triton X-100 in PBS for 30 min, blocked with 2% normal goat serum in PBS for 1 h at room temperature, and then incubated overnight at 4°C with primary antibodies: rabbit anti-c-fos (Abcam, 1:200) and guinea pig anti-vGlut1 (Millipore, 1:100). Sections were then incubated for 2 h at room temperature with the corresponding secondary antibodies: donkey anti-rabbit IgG conjugated with Alexa Fluor 594 (Invitrogen, 1:500) and goat anti-guinea pig IgG conjugated with tetraethyl rhodamine isothiocyanate (TRITC) (ImmunoReagents, 1:300). For negative controls, adjacent sections were treated with the same steps except by replacing the primary antibodies with PBS. Two symmetric images (×200) in the vlPAG area were acquired at the same exposure in each sight of 5–7 sections per subgroup using Nikon Eclipse 80i fluorescence microscope (Nikon, Japan).

#### Quantification and Statistical Analysis

Counting of FG-traced neurons, c-Fos- and vGlut1-labeled cells, and FG-traced neurons labeled by c-Fos or by vGlut1 was conducted on one of every 20 sections across the vlPAG, i.e., 5–7 sections per animal. On each section, two counting frames were symmetrically placed ventrolaterally to the midbrain aqueduct on images of × 200 magnification basing on the Rat Brain in Stereotaxic Coordinates (6th edition, Paxinos and Watson) (52). Cell counting was performed by two independent researchers to minimize counting bias. The results were recorded as the mean number of cells per frame. Fluorescence intensity of FG with and without c-Fos labeling within the frames mentioned above was measured separately, and those of FG with and without vGlut1 labeling measured separately using an Image J software (Image J 1.51n, USA). Here, thresholding background of the captured images for background subtracting was conducted. The measurements were made on 5–7 separate sections per animal, in 6 animals per group.

#### Statistical Analysis

All data were presented as mean ± standard error of the mean (M ± SEM). One-way analysis of variance (ANOVA) was applied to analyze the differences in cell number among groups and the differences between two groups for comparison. All statistical analyses were figured out by SPSS 16.0, and statistical significance was set at *P* < 0.05. Pairwise comparison was conducted by *post hoc* test after ANOVA, Tukey HSD, or Dunnett T3 depending on Test of Homogeneity of Variances. All histograms and graphs were performed with GraphPad Prism 6.02. Images were managed with Adobe Photoshop CS6.

## Results

### Analysis of Clinical Data

As shown in Table 1, there was no significant difference in age, gender, years of education, duration of epilepsy, and MMSE score among the three groups (*P* > 0.05). However, mean seizure frequency and HDRS scores in EwM group were higher than that in EwoM group (*P* < 0.05, Table 1).

### Group Differences in RsFC of the PAG

**Figure 2:**
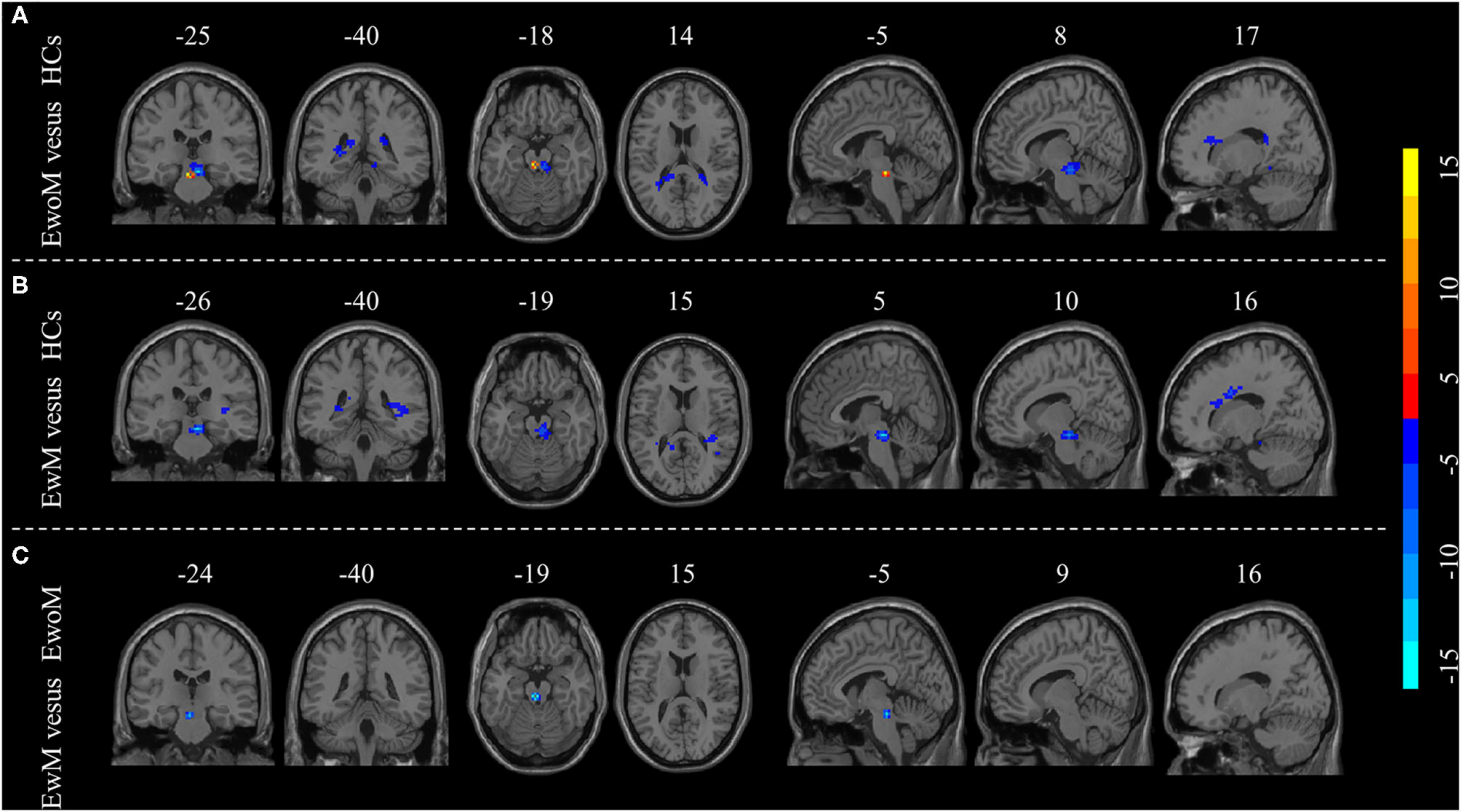
Group differences in resting-state functional connectivity (RsFC) to PAG. **(A)** Differences in RsFC to PAG between the EwoM group and HCs group; **(B)** Differences in RsFC to PAG between the EwM group and HCs group; **(C)** Differences in RsFC to PAG between the EwoM group and EwM group.

Compared to the HCs group, both EwoM and EwM groups showed decreased RsFC between PAG and left PPN (PAG-left PPN) and decreased RsFC between PAG and corpus callosum (CC) (PAG-CC), whereas PAG exhibited increased RsFC with the right PPN (PAG-right PPN) in EwoM group but not in EwM group. When comparing with EwoM group, EwM group exhibited decreased PAG-right PPN FC. However, we did not find significant difference in PAG-CC RsFC between EwM and EwoM groups. The significance of RsFC of PAG and corresponding significant clusters are showed in Table 2 and Figure 2.

**Table 2:** Regions with significant difference of RsFC between-groups.

**Brain region**	**MNI coordinate (x,y,z)**	**Voxel size**	***P*-value**
**EwoM vs. HCs**			
Left PPN	10, −23, −17	107	−6.624
Right PPN	−5, −30, −6	62	7.528
Corpus callosum	21, 17, 15	67	−5.749
**EwM vs. HCs**			
Left PPN	6, −25, −14	72	−6.912
Corpus callosum	25, 16, 10	59	−7.324
**EwM VS EwoM**			
Right PPN	3, 16, −9	26	−6.940

*HCs, healthy control; EwoM, epilepsy without migraine; EwM, epilepsy with comorbid migraine*.

### Correlation Analysis Between RsFC of PAG and Clinical Seizure Events

**Figure 3:**
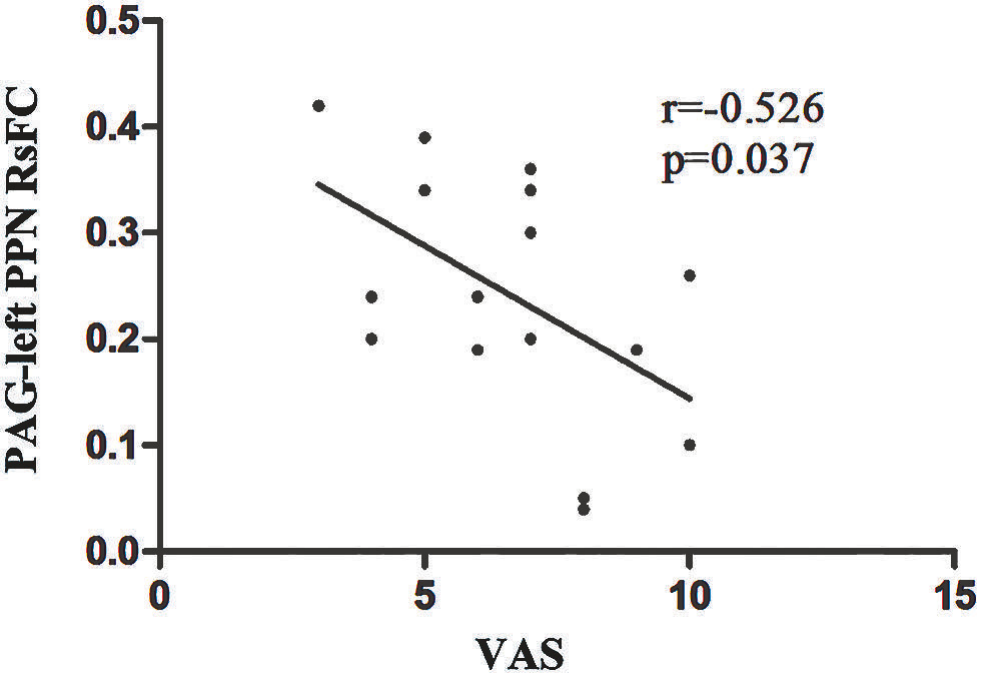
The relationship between PAG-left PPN RsFC and VAS scores.

**Figure 4:**
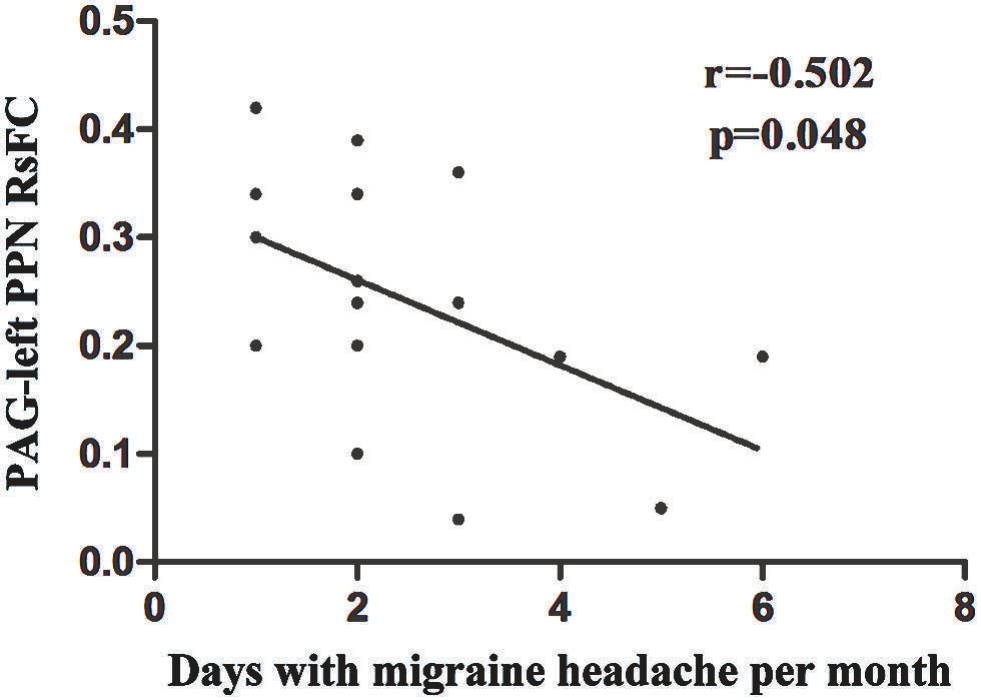
The relationship between PAG-left PPN RsFC and migraine frequency.

A significant negative correlation (*r* = −0.526, *P* = 0.037) was observed between VAS scores and the RsFC value of PAG-left PPN, where patients with lower RsFC value in this circuit had greater VAS scores (Figure 3). The RsFC value of PAG-left PPN was negatively correlated with the migraine attack frequency (*r* = −0.502, *P* = 0.048; Figure 4). In other words, patients with lower RsFC in this circuit had higher migraine attack frequency. In addition, our results did not find significant correlations between RsFC value of PAG-CC and migraine attack frequency (*r* = −0.048, *P* = 0.861) or with VAS scores (*r* = 0.339, *P* = 0.198).

### Alterations of FG-Traced Neurons Labeled by c-Fos After Seizure Stimulation in Rats With Chronic Epilepsy

**Figure 5:**
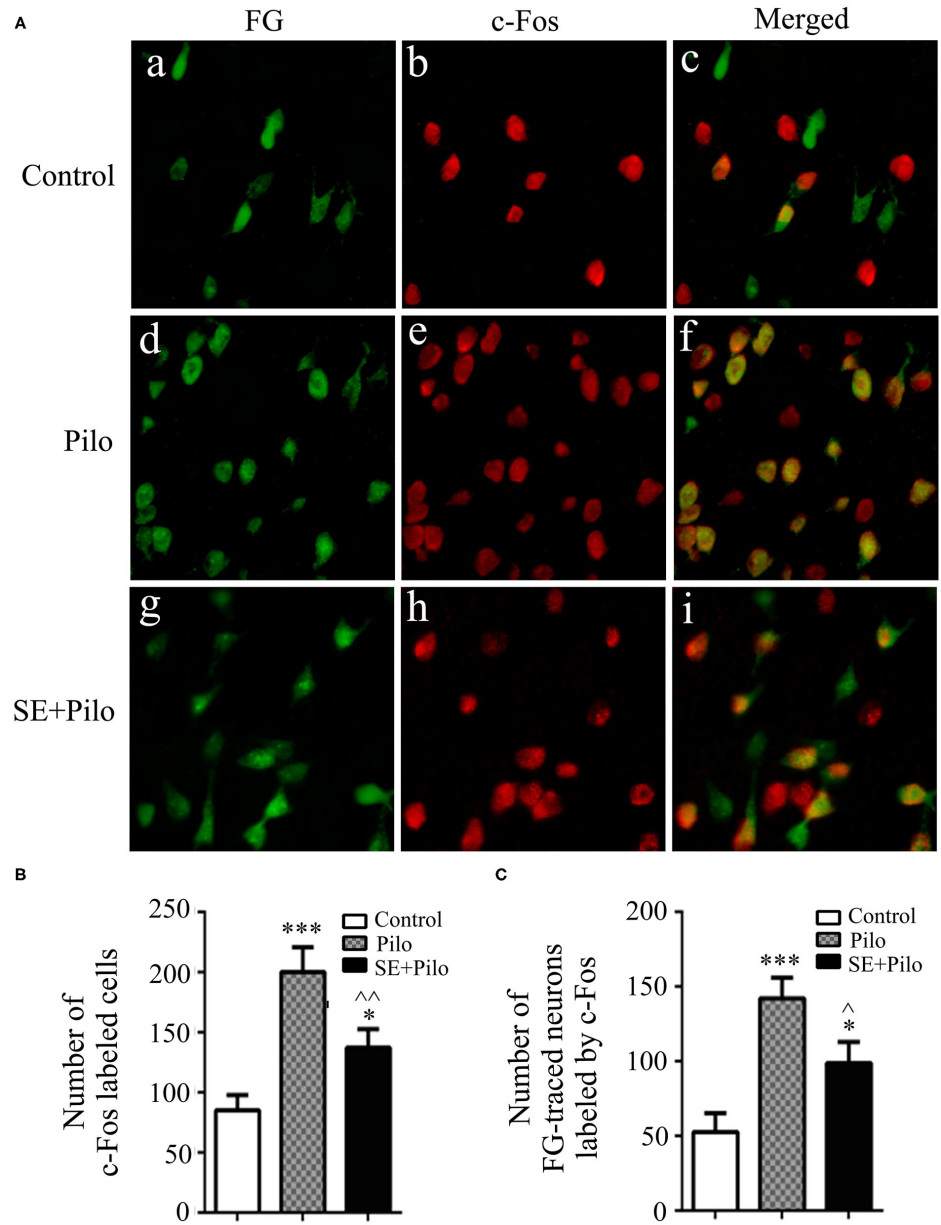
Neuronal activation induced by seizure stimulation in vlPAG is attenuated in chronic epilepsy. Coronal sections were immunostained for c-Fos (red) and retrogradely traced with FG (green) **(A)** The mean number of c-Fos immunoreactive cells after a seizure stimulation either in naïve rats (meshy bar) or in chronic epilepsy rats (solid bar) was significantly higher than that in non-stimulated control rats (open bar), but the mean number of c-Fos immunoreactive cells after a seizure stimulation in chronic epilepsy rats (solid bar) was significantly lower than that in naïve rats (meshy bar) **(B)** The mean number of c-Fos immunoreactive cells traced by FG after a seizure stimulation either in naïve rats (meshy bar) or in chronic epilepsy rats (solid bar) was significantly higher than that in non-stimulated control rats (open bar), but the mean number of c-Fos immunoreactive cells traced by FG after a seizure stimulation in chronic epilepsy rats (solid bar) was significantly lower than that in naïve rats (meshy bar) **(C)** A One-way ANOVA was performed. Different than the control group, ^*^P < 0.05, ^***^P < 0.001. Different than the Pilo group, ^∧∧^*P* < 0.01, ^∧^*P* < 0.05. *n* = 6 per group, scale bar = 100 μm.

The expression of c-Fos positive cells as well as FG traced cells in vlPAG were less in normal control group (Figure 5A). Seizure induced increase of c-Fos labeled cells in vlPAG both in naïve rats (Pilo group) and chronic epilepsy rats (SE+Pilo group) compared to control rats, but a significant lower increase in SE+Pilo group rats compared to Pilo group rats [Figure 5B, One-way ANOVA for comparison among groups, *F*_(2, 18)_= 12.135, *p* = 0.001; Pilo vs. control, *p* = 0.000; SE+Pilo vs. control, *p* = 0.042; SE+Pilo vs. Pilo, *p* = 0.007]. Seizure induced increase of c-Fos labeled cells traced by FG both in Pilo group and in SE+Pilo group rats, but a significant lower increase in SE+Pilo group rats compared to Pilo group rats [Figure 5C, One-way ANOVA for comparison among groups, *F*_(2, 18)_= 10.866, *p* = 0.001. Pilo vs. control, *p* = 0.000; SE+Pilo vs. control, *p* = 0.029; SE+Pilo vs. Pilo, *p* = 0.040]. This indicates that fewer vlPAG neurons projecting to PPN were activated after seizure stimulation in rats with chronic epilepsy as compared to that in normal control rats.

### Alterations of FG-Traced Neurons Labeled by vGlut1 After Seizure Stimulation in Rats With Chronic Epilepsy

**Figure 6:**
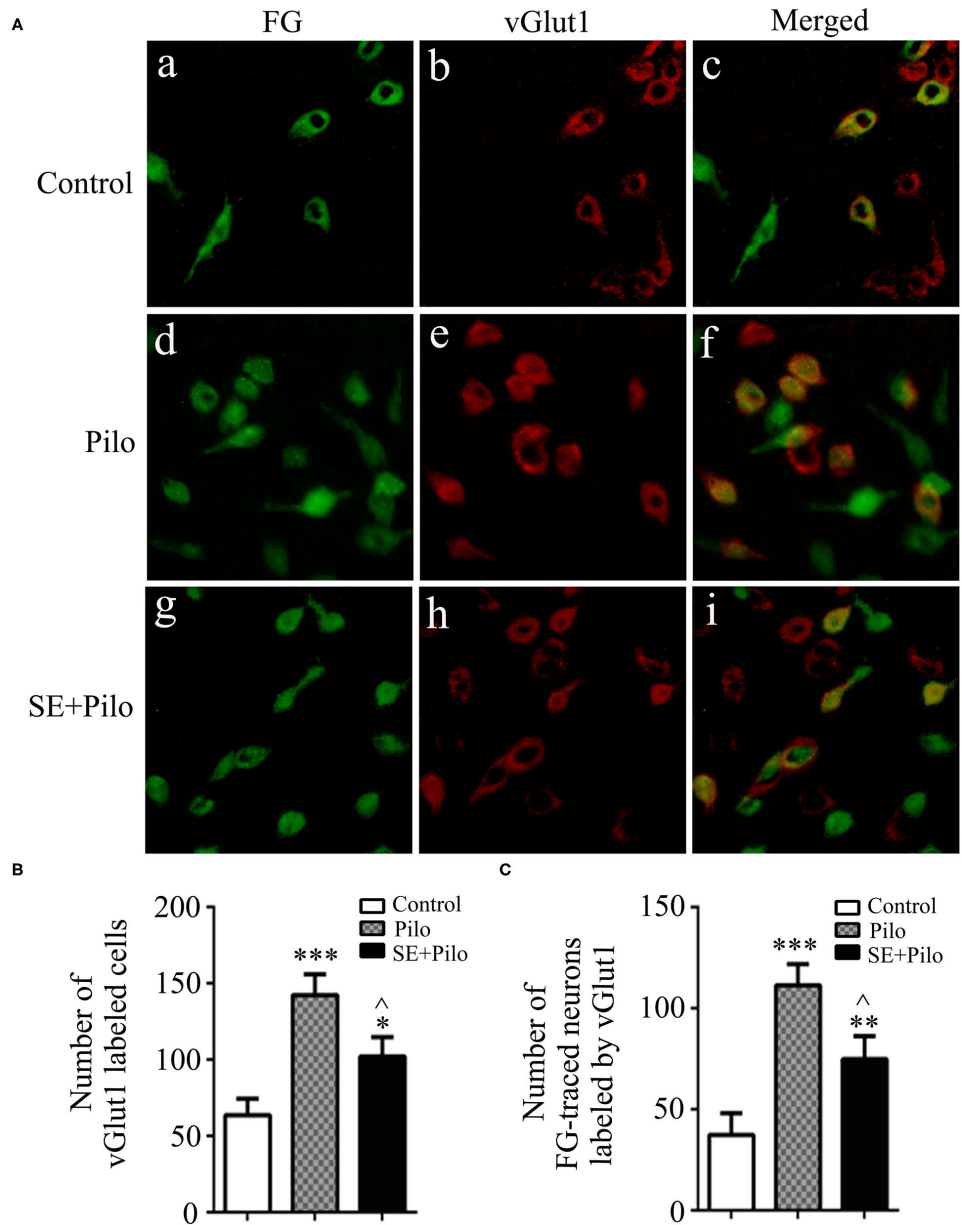
Excitatory transmission activation induced by seizure stimulation in vlPAG is attenuated in chronic epilepsy. Coronal sections were immunostained for vGlut1 (red) and retrogradely traced with FG (green) **(A)** The mean number of vGlut1 immunoreactive neurons after a seizure stimulation either in naïve rats (meshy bar) or in chronic epilepsy rats (solid bar) was significantly higher than that in non-stimulated control rats (open bar), but the mean number of vGlut1 immunoreactive neurons after a seizure stimulation in chronic epilepsy rats (solid bar) was significantly lower than that in naïve rats (meshy bar) **(B)** The mean number of vGlut1 immunoreactive neurons traced by FG after a seizure stimulation either in naïve rats (meshy bar) or in chronic epilepsy rats (solid bar) was significantly higher than that in non-stimulated control rats (open bar), but the mean number of vGlut1 immunoreactive neurons traced by FG after a seizure stimulation in chronic epilepsy rats (solid bar) was significantly lower than that in naïve rats (meshy bar) **(C)** A One-way ANOVA was performed. Different than the control group, *P < 0.05, **P < 0.01, ***P < 0.001. Different than the Pilo group, ^∧^*P* < 0.05. *n* = 6 per group, scale bar = 100 μm.

In normal control rats, few vGlut1 labeled cells presented in vlPAG, and few vGlut1 labeled neurons traced by FG presented (Figure 6A). Seizure induced increase of vGlut1 labeled cells in vlPAG both in naïve rats (Pilo group) and in chronic epilepsy rats (SE+Pilo group) compared to control rats, but a significant lower increase in SE+Pilo group rats compared to Pilo group rats [Figure 6B, One-way ANOVA for comparison among groups, F_(2, 18)_ = 9.907, *p* = 0.002; Pilo vs. control, *p* = 0.000; SE+Pilo vs. control, *p* < 0.046; SE+Pilo vs Pilo, *p* < 0.038]. Seizure induced increase of vGlut1 labeled cells traced by FG both in Pilo group and in SE+Pilo group rats, but a significant lower increase in SE+Pilo group rats compared to Pilo group rats (Figure 6C, One-way ANOVA for comparison among groups, *F*_(2, 18)_ = 11.542, *p* = 0.001. Pilo vs. control, *p* = 0.000; SE+Pilo vs. control, *p* = 0.028; SE+Pilo vs. Pilo, *p* = 0.032). This indicates that fewer vlPAG excitatory neurons projecting to PPN were activated after seizure stimulation in rats with chronic epilepsy as compared to that in normal control rats.

## Discussion

We are the first to demonstrate the functional alterations of PAG and its correlation with migraine in patients with epilepsy. We found that the FC of PAG-left PPN and PAG-CC was decreased in EwoM as well as in EwM group comparing with HCs group. An interesting phenomenon is that the PAG-right PPN FC was increased in EwoM group comparing with HCs group, which was not shown in EwM group. When comparing with EwoM group, EwM group exhibited decreased PAG-right PPN FC. Finally, the FC between PAG and PPN was negatively correlated with the attack frequency and VAS scores of migraine headache in epilepsy.

The migraine prevalence is significantly higher in patients with TLE than that in the general population (53). In fact, the interactive comorbid relationship between epilepsy and migraine has been well-noticed, but the underlying pathophysiological mechanism is not clear, though some possible shared mechanisms including genetic mechanism has been identified (54). For pain processing within brainstem, PAG is known to interact with many other brainstem nuclei such as the rostral ventromedial medulla and the subnucleus reticularis dorsalis that directly or indirectly project to the spinal dorsal horn and trigeminal nucleus (55). It is also demonstrated that decreased FC between PAG and other brain regions is involved in migraine headache basing on clinical neuroimaging study and experimental animal study (14, 56). In the current study, we found that the FC of PAG with other brain regions was decreased in TLE patients. Further, the FC of PAG was more remarkably decreased in TLE patients with comorbid migraine, and this FC was negatively correlated with the attack frequency and VAS scores of migraine headache. This indicates that the dysfunction of PAG network may be involved in the comorbidity of migraine in epilepsy.

The PPN, an elongated nucleus located in the rostral locomotor region of the brainstem, has functional afferent and efferent connections with multiple brain structures including PAG and structures outside of brainstem (27, 57). PPN has been shown to be crucial for the regulation of pain (58) as well as the regulation of seizure-induced hypoalgesia, a phenomenon of postictal antinociception due to the activation of PPN by seizure (28, 29). We demonstrated, for the first time, that the FC between PAG and left PPN is decreased during the interictal phase of seizures in patients with epilepsy. As the activation of PAG and PPN both have a pain suppressing effect, the decreased FC between PAG and PPN may give rise to a reduced pain threshold underlying migraine occurrence in epilepsy. Neuroimaging study has shown decreased activity in PAG in animals with epileptic seizures (59). This is consistent with our previous findings that there is a continuing loss of vlPAG neurons in rats with chronic epilepsy and neurochemical lesion to PAG decreases the pain threshold in epileptic rats (26). Furthermore, our current animal study showed that fewer vlPAG cells double-labeled with FG and c-Fos were activated in the chronic stage of epilepsy, indicating the decrease of excitatory transmission between PAG and PPN. The decreased FC between PAG and PPN in patients with TLE may be a representation of attenuated neural transmission from PAG to PPN in epilepsy rats. Previous studies have shown that both PAG and PPN activation have anti-epileptic effects (26, 60–62). Taken together, PAG and PPN both have antiepileptic and antinociceptive function, thus the decreased PAG-PPN functional connection might be proposed to be an underlying pathomechanism of the interactive comorbid relationship between epilepsy and migraine.

An interesting finding is the increased connectivity between PAG and the right PPN in patients without comorbid migraine but not in patients with comorbid migraine. This increased connectivity between PAG and right PPN seemed in conflict with the decreased connectivity between PAG and left PPN in patients without comorbidity of migraine. This discrepancy between the left and right PPN in connectivity with PAG might be due to the lateralization of seizure onset origin and a compensatory mechanism in epilepsy, though the sample size was not sufficient to analyze the influence of the lateralization of seizure onset origin. The finding that PAG exhibited increased RsFC with the right PPN in EwoM group but not in EwM group may also reflect the more generally favorable epilepsy outcome in the EwoM group of patients. This would be in line with the results from a recent work showing that TLE patients with increased level of connectivity had favorable outcome after anti-epileptic therapy (44).

CC connects the left and right cerebral hemispheres and mediates the interhemispheric transferring of signals, influencing on movements, sensory, emotion, behavior, cognition, memory, and complex integrative functions (63). In epilepsy, bilateral seizures with focal onset spread rapidly through CC and cause secondary structural damage related to demyelination in CC (64, 65). This is further supported by previous finding that decrement of CC connectivity is associated with disease history of TLE (66, 67). White matter including CC has been demonstrated with abnormalities in chronic pain including peripheral neuralgia and headache but less in episodic pain, indicating the CC abnormalities are the consequence of chronic pain attacking as the abnormalities are associated with pain intensity (68–70). This is further supported by the reversible CC lesion after headache attack in migraine patients (71–73). From literatures, the CC abnormalities are also associated with epilepsy as well as migraine attacks. Thus, the decreased PAG-CC connectivity is a logical accompaniment of epilepsy with or without comorbid migraine, though the decrement of the FC of PAG-CC was not correlated with the frequency of seizure or migraine attacks. In fact, this correlation cannot be excluded because of the limited size of the recruited patients in current study. The role of this decreased PAG-CC connectivity either in the development of migraine or in that of epilepsy is unknown, but this decreased connectivity might be associated with other clinical accompaniments of epilepsy and migraine, such as emotion, memory, sleep, etc., of which both PAG and CC are involved in the regulation (74–77).

In conclusion, an abnormal PAG neural network is present in TLE patients. It might be proposed that the decreased RsFC of PAG-PPN pathway is also associated with comorbidity of epilepsy and migraine. Thus, the disrupted connection between PAG and PPN may be an underlying pathophysiological mechanism for the interactive relationship between epilepsy and migraine.

## Limitations

Some limitations should be noted in this study. Firstly, the relatively few participants may limit the statistical power. For example, the lateralization of seizure onset origin cannot be used to analyze its association with lateral alteration of PAG-PPN connectivity and the correlation of PAG-CC connectivity with the frequency of seizure or migraine attacks. Thus, it is necessary to repeat our experiment by enlarging the sample size to better interpret the current results. Secondly, all the participants were on antiepileptic medication which may affect the FC as described in previous studies (78, 79). Thirdly, this is a cross-sectional study which cannot provide longitudinal alteration data of the participants, thus it cannot be determined whether the PAG functional alteration could be used to predict treatment outcomes in TLE patients comorbid with migraine. Fourthly, a group of patients with only migraine was not included in this study, and this might weaken the conclusion that the dysfunction of PAG network may play a causative role in the comorbidity of migraine in epilepsy.

## Data Availability Statement

The raw data supporting the conclusions of this article will be made available by the authors, without undue reservation.

## Ethics Statement

The studies involving human participants were reviewed and approved by the Ethics Committee of the First Affiliated Hospital of Anhui Medical University. The patients/participants provided their written informed consent to participate in this study. The animal study was reviewed and approved by the Ethics Committee of the First Affiliated Hospital of Anhui Medical University.

## Author Contributions

LW, X-TC, and YW made substantial contributions to conception and design of the study. LW, M-DZ, JZ, and Z-RD were responsible for subject recruitment, case diagnosis, and fMRI data acquisition and analysis. LW and X-TC were primarily responsible for animal experiments. LW, X-TC, and M-DZ contributed to statistical analysis and interpretation of data. LW, X-TC, and YW were responsible for drafting the manuscript and revising it. All authors approved the final version of the manuscript.

## Conflict of Interest

The authors declare that the research was conducted in the absence of any commercial or financial relationships that could be construed as a potential conflict of interest.

## Abbreviations

TLE: temporal lobe epilepsy
EwoM: epilepsy patients without migraine
EwM: epilepsy patients with comorbid migraine
PAG: periaqueductal gray
RsFC: resting-state functional connectivity
VAS: visual analog scale/score
PPN: pedunculopontine nucleus
CC: corpus callosum
rs-fMRI: resting-state functional magnetic resonance imaging
MMSE: mini-mental state examination
HDRS: Hamilton depression rating scale
ROI: region of interest.
